# Oxidative Stress, Inflammation, Gut Dysbiosis: What Can Polyphenols Do in Inflammatory Bowel Disease?

**DOI:** 10.3390/antiox12040967

**Published:** 2023-04-20

**Authors:** Lei Li, Peilan Peng, Ning Ding, Wenhui Jia, Canhua Huang, Yong Tang

**Affiliations:** 1 School of Basic Medical Sciences, Chengdu University of Traditional Chinese Medicine, Chengdu 611137, China; 2 West China School of Basic Medical Sciences and Forensic Medicine, State Key Laboratory of Biotherapy and Cancer Center, West China Hospital and Sichuan University and Collaborative Innovation Center for Biotherapy, Chengdu 610041, China; 3 School of Health and Rehabilitation, Chengdu University of Traditional Chinese Medicine, Chengdu 610075, China

**Keywords:** inflammatory bowel disease, oxidative stress, redox homeostasis, polyphenol, antioxidant, gut microbiota

## Abstract

Inflammatory bowel disease (IBD) is a long-term, progressive, and recurrent intestinal inflammatory disorder. The pathogenic mechanisms of IBD are multifaceted and associated with oxidative stress, unbalanced gut microbiota, and aberrant immune response. Indeed, oxidative stress can affect the progression and development of IBD by regulating the homeostasis of the gut microbiota and immune response. Therefore, redox-targeted therapy is a promising treatment option for IBD. Recent evidence has verified that Chinese herbal medicine (CHM)-derived polyphenols, natural antioxidants, are able to maintain redox equilibrium in the intestinal tract to prevent abnormal gut microbiota and radical inflammatory responses. Here, we provide a comprehensive perspective for implementing natural antioxidants as potential IBD candidate medications. In addition, we demonstrate novel technologies and stratagems for promoting the antioxidative properties of CHM-derived polyphenols, including novel delivery systems, chemical modifications, and combination strategies.

## 1. Introduction

**Figure 1:**
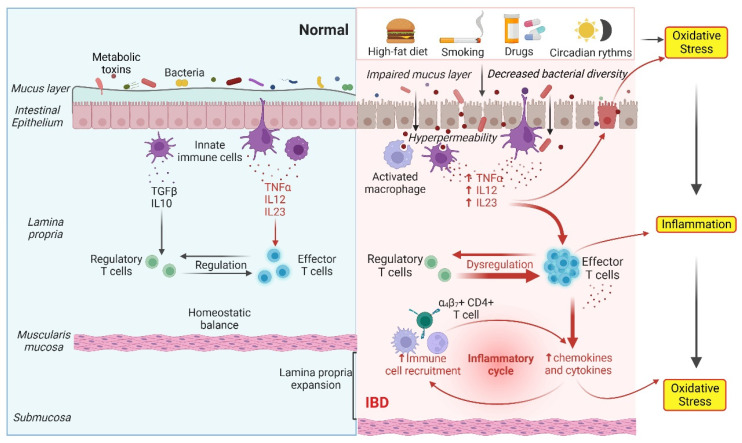
The pathogenic mechanisms of IBD. External pressures (high-fat diet, smoking, circadian rythms, and drug intervention) induce oxidative stress and can lead to gut dysbiosis and damage to the intestinal mucosal barrier, thus triggering a radical inflammatory response, which in turn stimulates the release of chemokines and cytokines, causing further oxidative stress and subsequent progression of IBD.

Inflammatory bowel disease (IBD), a chronic inflammatory disease primarily affecting the intestinal tract, includes Crohn’s disease (CD) and ulcerative colitis (UC) [1,2]. Both CD and DC share similar characteristics of intestinal mucosa inflammation caused by aberrant interaction between the immune response and the gut microbiota in genetically susceptible individuals [3]. The etiology of IBD remains elusive, but recent studies highlight the concept of an intriguing interface between oxidative stress, gut microbiota, and immune response [4,5]. Reactive oxygen species (ROS), the hallmark and executor of oxidative stress, exert dual functions to affect the progression of cells and tissues [6,7]. Moderate ROS (at physiological levels in the body) can act as second messengers to regulate cellular physiological processes involving endogenous homeostasis maintenance and biological functions such as redox signal transduction, gene expression, and receptor activation, which is beneficial for tissue turnover and cell proliferation [8,9]. Excessive ROS (above the toxic threshold) can damage cellular molecules such as DNA, proteins, and lipids, thus causing cell senescence or death [10]. In general, the occurrence and development of IBD are accompanied by persistent oxidative stress and inflammatory responses induced by dysbiosis of the gut microbiota [11,12]. A high-fat diet, smoking, circadian rhythms, and drug intervention can disturb the intestinal microbiota [13]. This plays a crucial role in host immune responses, reducing bacterial diversity, inhibiting gut microbiota regulatory properties, and raising intestinal ROS [14]. These excessive ROS can act as upstream stimuli to evoke aberrant activation of the intestinal immune system, causing damage to the intestinal mucosal barrier, including a decrease in mucous secretion, antimicrobial peptide secretion, and the destruction of tight junctions [15]. Importantly, radical immune cells activate inflammatory signaling pathways such as NF-κB, JAK/STAT, and MAPK signaling pathways [16,17,18], promote the release of proinflammatory factors (e.g., TNF-α, IL-6, and IL-1β) and oxidases (e.g., iNOS, COX-2, and NOX [19,20]), and further increase the level of oxidative stress in the intestinal tract [21], which will remold the gut microbiota and form a loop of oxidative stress–ROS–inflammation–ROS–oxidative stress (Figure 1). Accordingly, such a loop can be used as a potential therapeutic target to avoid the occurrence and progression of intestinal inflammation by alleviating oxidative stress in the intestine and reducing the stimulation of gut microbiota and immune cells by ROS, thereby recovering the intestinal mucosal barrier.

Current therapeutics for IBD treatment have focused on targeting the immune system and intestinal bacteria. For example, monoclonal antibodies (such as infliximab, ustekinumab, and risankizumab) have been used to neutralize proinflammatory cytokines [22,23,24], and immune cell infiltration inhibitors (e.g., ozanimod) [25] or inflammatory signaling pathway inhibitors (e.g., tofacitinib) have been used to attenuate the migration of lymphocytes and block the JAK/STAT signaling pathway [26]. Some sphingolipids, such as SphK1 inhibitors and S1PRs antagonists have been developed as novel anti-inflammatory agents [27]. Additionally, corresponding studies have found that probiotics and prebiotics, e.g., *Bifidobacterium*, *Lactobacillus*, and fructooligosaccharides, can alter the composition of intestinal bacteria, which is also beneficial for the attenuation of inflammation and oxidative stress [28,29,30]. Indeed, all these therapeutic options generate positive effects in patients with IBD. However, limitations such as poor response, systemic side effects, high recurrence rate, and high cost still need to be addressed [31,32]. Recently, an increasing number of studies have applied antioxidants to alleviate oxidative stress and inflammation in the intestinal tract and have received some favorable feedback for the treatment of IBD [33]. Therefore, exploiting pathogenesis-based therapeutics remains attractive for new drug discovery and development.

**Figure 2:**
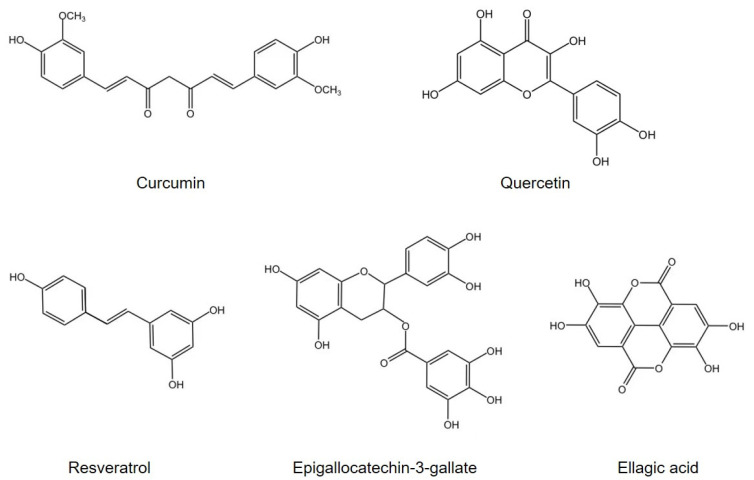
The structure of polyphenols in this review (curcumin, quercetin, resveratrol, epigallocatechin-3-gallate, and ellagic acid). This figure was created with Kingdraw.

Polyphenols, a broad family of natural products, are the most common natural antioxidants and are characterized by multiple phenolic units in their structures [34,35]. Polyphenols can be classified into four major categories: flavonoids, phenolic acids, lignans, and stilbenes [36,37]. The chemical structures of some of the polyphenols are depicted in Figure 2. Natural polyphenols are commonly present in many plants, such as dietary sources (e.g., vegetables, fruits, spices, and plant-derived beverages) and herbal medicines, especially Chinese herbal medicines (CHMs), which share the holistic principles of “homology of medicine and food” found in traditional Chinese medicine (TCM) and have versatile biological properties [38,39]. Due to the hydroxyl group in their structure, polyphenols are endowed with inherent antioxidant activity, allowing them to scavenge radicals by hydrogen atom transfer or single-electron transfer reactions [40,41]. In recent years, multiple studies have confirmed the antioxidant and anti-inflammatory activities of polyphenols in IBD [42]. However, the limitations of polyphenols in delivery, stability, and targeting impede their further preclinical research and clinical evaluations. Therefore, recent studies have focused on optimizing the application of polyphenols using modification or nanotechnology.

Our search approach was centered on the utilization of several public databases, namely, PubMed, ClinicalTrials, Google Scholar, and Web of Science, for sourcing data available up to April 2023. The search terms employed included Inflammatory Bowel Disease, Oxidative Stress, Redox, ROS, Gut Dysbiosis, Gut Microbiota, and Polyphenols. The selection process entailed the application of specific inclusion and exclusion criteria. The inclusion criteria were as follows: (1) only papers published in the English language and within the last 20 years; (2) articles with a high citation rate; (3) studies with broad application prospects. On the other hand, the exclusion criteria were as follows: (1) articles with low credibility ratings; (2) papers published in languages other than English; (3) articles that were not available in full-text format. In this review, we summarize the underlying therapeutic mechanisms of polyphenols for modulating redox homeostasis to repair the intestinal mucosal barrier and focus on the application of polyphenols as potential protective and therapeutic drugs in IBD treatment based on redox-targeted therapeutics. In addition, we also discuss novel technologies or methods for overcoming the limitations of polyphenols for effective IBD treatment and prevention.

## 2. Gut Dysbiosis Causes IBD through Oxidative Stress and the Inflammatory Response

The gut microbiota in the human intestinal tract has a rich diversity and contains more than 1000 species [43,44], 90% of which are composed of four major phyla: Firmicutes (49–76%), Bacteroidetes (16–23%), Proteobacteria, and Actinobacteria [45,46]. Gut microbiota plays a range of roles in mineral absorption and carbohydrate degradation [47,48], as well as the synthesis of amino acids and vitamins [49,50], all of which are essential for the hosts’ biological function [51]. The immunomodulatory effects of the gut microbiota are mainly expressed in the synthetic pathway of short-chain fatty acids (SCFAs) and the metabolism of tryptophan, bile acid, succinate, and sphingolipids.

Briefly, the gut microbiota can metabolize dietary fiber into SCFAs [52], such as butyrate, propionate, and acetate, which can enhance mucosal immunity tolerance and maintain the intestinal barrier function by activating G protein-coupled receptors (GPCRs) and, subsequently, T regulatory cells (Tregs) [53,54]. In addition, indoles and their derivatives, which are tryptophan metabolites produced by the intestinal commensal *Clostridium sporogenes*, can regulate intestinal inflammation by activating the aryl hydrocarbon receptor [55]. Interestingly, some specific indole derivatives, such as indoleacrylic acid, can lead to mucin gene expression and stimulate the Nrf2 signaling pathway, thus increasing the antioxidant ability of the intestinal tract [56]. Moreover, the gut microbiota promotes bile acid metabolism, resulting in secondary bile acids that can be sensed by the farnesoid X receptor (FXR) and Takeda G protein-coupled receptor 5 (TGR5), and induces gene expression related to microbial defense, immune cell maturation, and cytokine release [57,58]. Similarly, succinate, the fuel for butyrate and ATP production, is a metabolite of the *Bacteroides* species with dietary fiber [59]. Importantly, it is able to drive IL-1β production by stabilizing hypoxia-inducible factor-1α (HIF-1α) and activating dendritic cells to promote inflammatory progression [60]. Sphingolipids, the most notable ceramide, can be synthesized by Bacteroidetes via bacterial serine-palmitoyl transferase, and they can act as signaling molecules to modulate inflammatory pathways and lead to resolution of inflammation in host cells [61,62,63]. By contrast, the immunomodulatory function of the gut microbiota is significantly different in IBD.

The host provides an indispensable nutrient environment for the gut microbiota, which in turn feeds back essential vitamins, neurotransmitters, and SCFAs to form a symbiotic relationship [64]. Notably, decreased gut microbial diversity and dysbiosis are hallmarks in patients with IBD. Related research has indicated that the proportions of Firmicutes and Bacteroidetes are significantly reduced in IBD patients, while the ratio of Proteobacteria is increased [65]. Similarly, metagenomics sequencing of gut microbes also demonstrated that the microbial diversity in IBD patients is obviously attenuated compared to that in healthy individuals, especially beneficial bacteria, such as *Bacteroides*, *Lactobacillus*, and *Eubacterium*, causing oxidative stress and leading to gut dysbiosis and inflammation [66,67]. In parallel, some incremental pathogenic bacteria, such as adherent invasive *Escherichia coli* (*E. coli*) [68], can lead to damage to the intestinal mucosal epithelium and enhance intestinal permeability, causing gut metabolic wastes (e.g., cadaverine, taurine) and bacterial metabolic toxins, such as lipopolysaccharide (LPS), to permeate into the blood and eventually aggravating IBD [69,70,71]. In summary, therapeutic strategies that restore gut microbiota homeostasis by regulating the ratio of beneficial bacteria to pathogenic bacteria and relieving oxidative stress and inflammation have great promise.

## 3. Polyphenols Retard Oxidative Stress and Inflammation via Modulation of Gut Microbiota

**Figure 3:**
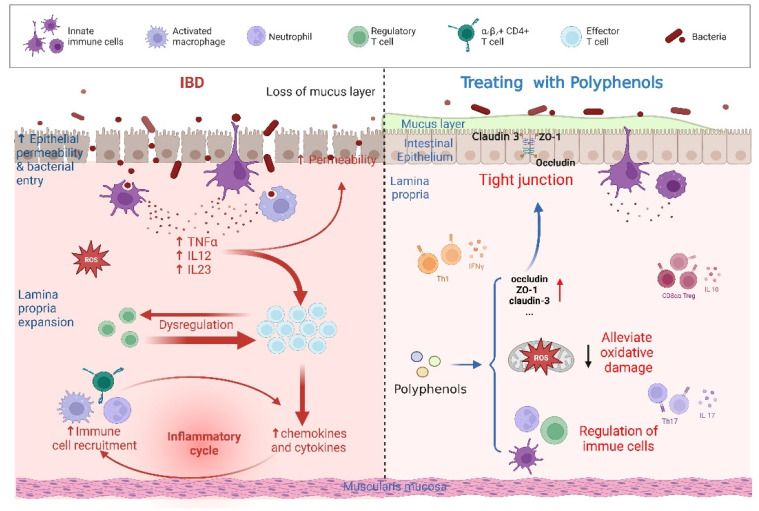
Polyphenols retard inflammation and restore redox homeostasis of the gut by regulating microbiota. Polyphenol intervention increases the diversity of gut microbiota, which is essential for maintaining the intensity of the intestinal mucosal barrier, thus promoting the antioxidative defense through activating Nrf2, elevating the content of SOD and GSH, and weakening the activity of NF-κB. In this process, the gut microbiota metabolite SCFAs assist differentiation of Treg cells, counteracting excessive inflammation caused by activation of effector T cells.

Prebiotics and probiotics, the conventional adjuvants in treating IBD, have been widely used to restore gut microbiota diversity. Interestingly, polyphenols have prebiotic-like properties that enhance the relative abundance of beneficial bacteria, strengthening their antioxidant capability [72,73]. In addition, the metabolites of polyphenols in the colon segment catabolized by microbiota results in increased anti-inflammatory activity [74,75]. A great number of studies have shown that polyphenolic compounds positively regulate antioxidant signaling pathways, such as Nrf2 [76,77], to intensify the intestinal mucosal barrier, which is also beneficial for the homeostasis of the gut microbiota [78,79,80]. Intriguingly, some microbial phenolic metabolites, which are generated through the interaction of undigested phenolic compounds and the microbiota found in the colon, have greater relevance and bioavailability than their precursors [12]. In this section, polyphenolic compounds such as curcumin, quercetin, resveratrol, and epigallocatechin-3-gallate (EGCG) or corresponding microbial metabolites will be reviewed for the underlying mechanisms which can modulate the gut microbiota for IBD treatment (Figure 3).

### 3.1. Curcumin

Curcumin, a widely applied natural polyphenol mainly derived from CHMs (e.g., *Crocus sativus* L., and *Curcuma longa* L.) [81,82], has been reported to have promising pharmacologic activities in IBD therapy [83]. The antioxidant, anti-inflammatory, and antimicrobial properties of curcumin endow it with an enormous advantage in modulating the gut microbiota and alleviating oxidative stress and inflammation, thus protecting the intestinal mucosal barrier. In the clinical setting, curcumin increases approximately 69% of detected gut bacterial species in the human gastrointestinal tract, such as *Clostridium* and *Bacteroides* [84]. In experimental animals, curcumin can promote the diversity and relative abundance of gut microbiota in mice with UC, which potentially weakens the activity of Th17 cells (proinflammation) and enhances the response of Treg cells (anti-inflammation) [85]. A curcumin-supplemented diet can also improve gut bacterial homeostasis, including maintaining the abundance of *Lactobacillales* and decreasing the proportion of *Coriobacterales* in azoxymethane (AOM)-induced persistent colitis [86]. Interestingly, curcumin can increase butyrate levels by modulating the gut microbial structure, therefore expanding the ratio of regulatory T cells and dendritic cells in the colonic mucosa and inhibiting the onset of IBD [87]. A recent report exhibited multiple protective effects of curcumin by modulating the gut microbiota. Thus, oral administration of curcumin restored impaired bacterial diversity caused by dextran sulfate sodium (DSS), enhanced the expression of tight junction proteins such as occludin and ZO-1, promoted the activity of the antioxidase superoxide dismutase (SOD), and inhibited the MAPK/NF-κB/STAT3 pathway and caspase-3-induced apoptosis [88].

### 3.2. Quercetin

Quercetin has been shown to have excellent modulation potential on bacteria in many types of diseases, including neurodegenerative disease, metabolic disease, and IBD [89,90,91,92]. As an important constituent of CHM-derived polyphenols, quercetin prevents the onset of IBD by inhibiting oxidative stress and inflammation, in which gut bacteria are indispensable players. For example, quercetin has been identified as having a protective effect on Caco2 cells against hydrogen peroxide (H_2_O_2_)-induced oxidative damage by elevating intracellular glutathione (GSH) content. This is in concordance with the in vivo model [93]. Similarly, a study reported that quercetin could ameliorate DSS-induced colitis in a mouse model by remodeling macrophages’ proinflammatory activity via a heme oxygenase-1 (HO-1)-dependent pathway and maintaining gut microbial diversity and commensal microbe homeostasis [94]. DSS-induced colitis can enhance the activity of myeloperoxidase (MPO) and malondialdehyde (MDA), reduce GSH, and downregulate gut microbiota diversity, whereas quercetin administration can attenuate oxidative stress in the intestinal tract and increase gut microbial diversity [95]. In general, the administration of quercetin can improve the abundance of beneficial bacteria such as *Bacteroides*, *Bifidobacterium*, *Clostridium*, and *Lactobacillus*, and in turn, it distinctly weakens that of *Enterococcus* and *Fusobacterium* [96]. Quercetin exerts a profound impact on the diversity of the gut microbiota, while the gut microbiota also influences the production and degradation of quercetin. For example, the gut microbiota express β-glucosidase, which has the ability to efficiently deglycosylate lignans and flavonoids [97]. Some specific bacterial strains, such as *Clostridium* and *Bacteroides*, are able to induce cycloreversion of quercetin and release critical microbial metabolites (e.g., 3,4-dihydroxyphenyl acetic acid and protocatechuic acid) [98], which also act as critical players in the progression of IBD [99,100].

### 3.3. Resveratrol

Resveratrol is a well-known polyphenolic compound extracted at high levels from grape skins, peanuts, blueberries, and the roots of some CHMs, especially *Polygonum cuspidatum*, *Morus alba* L., and *Panax notoginseng* (Burkill) [101,102]. Due to its multiple anti-inflammatory, antioxidant, antitumor, and antimicrobial biological effects, resveratrol has been proven beneficial in various diseases, such as diabetes mellitus, obesity, cancer, and IBD [103,104,105]. The antimicrobial activity of resveratrol could impair the homeostasis of gut microbiota. Recent evidence has confirmed that resveratrol can interact with the gut microbiota in vitro and in vivo to acquire essential pharmacological activities, including antioxidant and anti-inflammatory capabilities [106]. In addition, resveratrol administration can regulate intestinal bacteria composition resulting in therapeutic potential. According to a corresponding study, resveratrol treatment can restore 2,4,6-trinitrobenzenesulfonic acid (TNBS)-induced murine colitis by increasing the ratio of *Bacteroides acidifaciens*, while downregulating *Ruminococcus gnavus* and *Akkermansia mucinphilia* and reducing SCFAs to homeostatic levels [107]. Likewise, resveratrol treatment can lead to an anti-inflammatory T cell response by altering the gut microbiome and SCFAs, eliminating DSS-induced inflammation, and preventing inflammation-mediated colorectal cancer [108]. As a universal dietary supplement, whether resveratrol can exert pharmacological activity in vivo at an attainable dietary dose is of great importance. In a DSS-induced colitis rat model, a low dose of dietary resveratrol mitigates intestinal oxidative stress and inflammation and restores beneficial bacteria such as *lactobacilli* and *bifidobacteria* [109]. In a clinical setting, supplementation with resveratrol in patients with UC is efficacious in reducing plasma levels of TNF-α, high sensitivity-CRP, and NF-κB activity compared to a placebo [110]. Additionally, the microbial metabolites of resveratrol, specifically 3-(4-hydroxyphenyl)-propionic acid, have been shown to effectively attenuate the inflammatory responses of DSS-induced colitis. This result provides new insights to understand the protective effects of resveratrol against IBD [106].

### 3.4. Other Agents

Many other polyphenolic compounds can be effective in IBD treatment and prevention by regulating the gut microbiota. For example, green tea is one of the most consumed beverages and widely used CHMs worldwide. It contains abundant tea polyphenols, such as epigallocatechin, epicatechin-3-gallate, epicatechin, and EGCG [111,112]. Notably, tea polyphenols, especially EGCG, have attracted significant attention for delaying aging, reducing cholesterol, and preventing cancer due to their outstanding antioxidative ability. EGCG has been reported to reinforce GSH production and SOD activity, decrease the content of NO and malonaldehyde (MDA), and restrain TNF-α, IFN-γ, and NF-κB production to relieve the inflammatory status of the intestinal mucosa [113,114]. In fact, oral EGCG can attenuate colitis in a gut microbiota-dependent manner by enhanced *Akkermansia* abundance and butyrate production [115]. Similarly, ellagic acid (EA), a natural polyphenol found in pomegranate (*Punica granatum* L.) peel, has shown anti-inflammatory and antioxidant properties in many disorders. EA can regulate the NF-κB signaling pathway, intimately related to inflammation and oxidative stress, to decrease the expression of COX-2 and iNOS and phosphorylated MAPKs and impair nuclear NF-κB translocation, thus assuaging chronic colonic inflammation and oxidative stress in rats [116]. Meanwhile, EA has been identified to regulate the microbiome to prevent infectious colitis [117,118] and has also been applied in IBD prevention by reducing *Streptococcus* abundance in the gut [119]. In addition, urolithin A, a microbial metabolite of EA, exhibits antioxidant and anti-inflammatory effects by regulating the Nrf2 pathway, thereby enhancing the gut barrier integrity [120]. (Table 1).

## 4. Inflammatory Response and Oxidative Stress in IBD

According to the above findings, oxidative stress and unbalanced gut microbiota are the most significant phenotypes in the occurrence and progression of IBD. However, inappropriate inflammatory responses also play a core role in IBD pathogenesis. The initiation of the inflammatory response requires specific stimuli, such as pathogens and antigens, to activate the host defense. Innate immune cells such as dendritic cells and macrophages can recognize invariant microbial molecular patterns (pathogen-associated molecular patterns (PAMPs)) and endogenous damage-associated molecular patterns (DAMPs) through germline-encoded pattern recognition receptors (PRRs), including Toll-like receptors (TLRs) and nucleotide oligomerization domains (NODs), to distinguish “self” from “nonself” [121,122,123]. The occurrence of IBD is based on the recognition of gut microbiota-related PAMPs and oxidative stress-related DAMPs and the subsequent inflammatory response. For example, gut microbiota-originated metabolic toxins, such as LPS, can act as PAMPs to activate immune cells [123,124]. In parallel, drug-, smoking-, and circadian rhythm-induced oxidative stress can damage intestinal epithelial cells (IECs), such as Paneth cells and goblet cells, releasing cell debris, calreticulin, heat shock proteins (HSPs) and ATP, which can act as DAMPs to promote the progression of inflammation and subsequent pyroptosis and necroptosis [125,126,127,128]. Immune cells secrete products that are actively involved in the initiation and preservation of inflammation, leading to gut tissue damage.

Many immune tissue cells reside in the different layers of the intestinal tract, acting as guardians for intestinal immune homeostasis [128,129]. The gatekeepers, macrophages, are not homogenous but have two distinct subsets: proinflammatory (M1) macrophages and anti-inflammatory (M2) macrophages [130]. M1 macrophages secrete high levels of proinflammatory cytokines such as IL-1β, IL-6, IL-8, IL-12, and TNF, which can produce high levels of ROS to trigger further inflammation and disrupt the intestinal epithelial barrier [131,132]. Conversely, M2 macrophages produce significant amounts of anti-inflammatory cytokines, including IL-10, TGF-β, EGF, and VEGF, and promote the resolution of inflammation and recovery of the intestinal epithelial barrier [129]. Therefore, stimulating macrophage M2 polarization is a potential strategy for IBD treatment. IECs and macrophages can release IL-1α during inflammation, which recruits neutrophils that upregulate PRRs to fight inflammogens [133]. Neutrophils can phagocytize and eliminate pathogens through ROS from the respiratory burst and release extracellular neutrophil traps (NETs), which produce the bactericidal oxidant hypochlorous acid (HOCl), causing tissue damage when unregulated [133,134,135]. Dendritic cells (DCs) are the most important antigen-presenting cells (APCs) and can link innate and adaptive immunity through their phenotypic heterogeneity and functional plasticity. In IBD, PAMPs and DAMPs can be recognized by DCs with high RPPs expression, sequentially activating naive T cells, stimulating proliferation, and thus impairing gut barrier function [136,137,138]. T cells comprise the largest proportion of immune cells and have two main subgroups, CD4+ T cells and CD8+ T cells, in which CD8+ T cells exert cytotoxic functions [139]. CD4 T cells can be divided into effector and regulatory cells (Tregs). The ratio of Treg/Th17 affects the outcomes of inflammation [140]. As the most important counterbalance to the proinflammatory environment of the immune system, Treg cells secrete cytokines such as TGF-β and IL-10 to mediate tissue repair and epithelial cell differentiation and interact with APCs to suppress inflammation and maintain intestinal homeostasis [141,142]. These immune cells are triggered by DAMPs or PAMPs and interact with varying function, leading to alterations in the immune tolerance environment, thereby causing persistent oxidative stress and inflammation and subsequent tumorigenesis in the gut [143,144].

As mentioned above, immunomodulators, monoclonal antibodies, and corticosteroids, which affect proinflammatory cytokines and immune cells, have been routinely used as therapeutics for IBD treatment. However, poor response and systemic side effects still limit the development of curative treatment schemes and impair their therapeutic effect.

## 5. Polyphenols Regulate the Immune Response and Oxidative Stress to Restrain IBD

**Figure 4:**
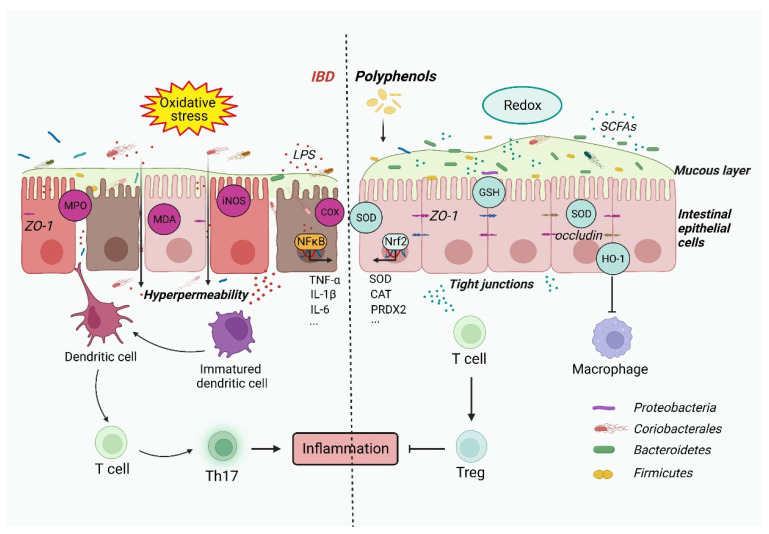
Polyphenols regulate immune response and oxidative stress to treat IBD. Polyphenol treatment reinforces the intestinal mucosal barrier by enhancing the tight junction proteins. In addition, polyphenols can eliminate ROS and evoke antioxidative signaling pathways to alleviate oxidative damage. Similarly, administration with polyphenols can regulate the differentiation of immune cells and attenuate active immune response, thus inducing IBD remission.

Polyphenols have exhibited excellent pharmacodynamic functions, including antioxidant and anti-inflammatory functions, by reducing oxidative stress, regulating the Nrf2 and NF-κB signaling pathways, and modulating the immune system [76,145,146]. Recent evidence has also shown that some polyphenols exert direct effects on supporting the intestinal mucosal barrier integrity. Interestingly, it seems that natural polyphenolic compounds influence all pivotal mechanisms involved in the pathogenesis of IBD [31]. An increasing number of studies have verified that natural polyphenols effectively alleviate the severity of intestinal inflammation and oxidative stress during the onset of IBD. Exploiting such antioxidant- and anti-inflammation-based therapeutics using polyphenols is favorable for patients with IBD. In this section, some polyphenols, including curcumin, quercetin, resveratrol, and EGCG, will be discussed for their potential therapeutic effect through the regulation of oxidative stress and inflammation in IBD prevention and treatment. (Figure 4).

### 5.1. Curcumin

Dietary curcumin has been suggested as a daily adjuvant to maintain body health. In IBD, the pharmacologic properties of curcumin not only modulate the gut microbiota but also promote homeostasis between oxidative stress and inflammation. Primarily, curcumin is able to enhance the expression of tight junction proteins, such as occludin, ZO-1, and claudin-3, and maintain the integrity of the intestinal mucosal barrier by preventing endoplasmic reticulum stress-mediated IEC apoptosis [31,147]. In addition, a recent study verified that curcumin could impair ROS (H_2_O_2_)-induced oxidative damage by stimulating the heme oxygenase-1 (HO-1) signaling pathway [147]. Curcumin is also capable of affecting the outcomes of inflammation by altering the status of immune cells. For example, curcumin modulates the differentiation of naive T cells and changes the ratio of central memory T (T_CM_) cells and effector memory (T_EM_) cells in experimental colitis, which downregulates the levels of proinflammatory cytokines such as IL-7, IL-15, and IL-21 by inhibiting the JAK1/STAT5 signaling pathway [148]. Likewise, another study demonstrated that curcumin could regulate the balance of follicular helper T cells (Tfh) and follicular regulatory T cells (Tfr) in the intestinal tract by suppressing the synthesis of IL-21 [149]. As mentioned above, macrophages can produce ROS to accelerate the development of intestinal inflammation, so targeting macrophages may be a potential strategy to eliminate oxidative stress and inflammation. In a recent study, curcumin intervention was found to reduce the concentration of IL-1β, as well as the production of intracellular ROS in macrophages [149]. Furthermore, curcumin supplementation can change macrophage polarization from M1 to M2 and decrease the expression of PRRs such as TLR2, TLR4, and NF-κB in the colon tissue of mice with IBD [150]. Accordingly, these results illustrate the dual function of curcumin in restraining oxidative stress and inflammation in IBD treatment.

### 5.2. Quercetin

Quercetin has been investigated as a significant adjuvant for IBD treatment or prevention due to its acknowledged antioxidant and anti-inflammatory characteristics. Oral administration of quercetin works on the intestinal mucosa, thus exerting its therapeutic effects. Quercetin can strengthen the intestinal mucosal barrier by promoting the expression of tight junction proteins, such as ZO-1 and claudin-1, thereby attenuating the hyperpermeability of the intestine during the development of IBD [151,152]. In addition, quercetin can promote the proliferation of intestinal cells [93] and increase the population of goblet cells through the activation of the PKCα/ERK1-2 signaling pathway to upregulate mucus production and secretory capacity [153]. Oxidative stress clearly can accelerate the development of IBD and exacerbate clinical symptoms [93]. Quercetin is able to promote the synthesis of GSH and the expression of Nrf2, which are efficient in abrogating oxidative stress-induced lipid peroxides and elevating MPO, MDA, and NO [95,154]. As detailed above, the infiltration of immune cells stimulates the exacerbation of IBD. Quercetin can impair the recruitment of neutrophils and reduce the infiltration of neutrophils and macrophages [152], which may produce ROS and induce oxidative stress and inflammation in the colon tissue of IBD patients [135]. Surprisingly, in colitis, quercetin promotes M2 macrophage polarization to decrease the secretion of proinflammatory cytokines such as TNF-α, IL-23, and IL-12 in colonic tissue [94]. Moreover, quercetin can affect some transcription factors, such as CCAAT/enhancer-binding protein β (C/EBP-β), to inhibit the production of downstream cytokines, including TNF-α and IL-6, in dendritic cells, thereby attenuating colitis in mouse models of IBD [155]. The ratio of Th17/Treg cells changes after oral administration of quercetin, in which enhanced Treg cells restrain the development of IBD [152].

### 5.3. Resveratrol

Resveratrol has the potential to improve the intestinal mucosal barrier. Evidence suggests that resveratrol can suppress IECs apoptosis while maintaining colonic mucosal architecture, perhaps sustaining the intestinal mucosal barrier [109,156]. Tight junction proteins such as occludin, claudin, and ZO-1 in colon tissue can be impaired at the onset of IBD, but resveratrol intervention can restore their expression to enhance the intestinal mucosal barrier, which maintains gut homeostasis [157]. Correspondingly, resveratrol modulates the antioxidant defense system to retard IBD [158]. For example, resveratrol can reinstate redox homeostasis in colon tissue by preventing lipid peroxide and glutathione depletion in TNBS-induced ulcerative colitis. Similarly, an analog of resveratrol has been shown to have a regulatory effect on Nrf2, which can strengthen the antioxidant property of the intestinal tract against DSS-induced inflammatory damage [158]. As described in this section, resveratrol favors the intestinal mucosal barrier. Indeed, resveratrol attenuates the recruitment and infiltration of neutrophils into colon tissue, which may be attributed to enhanced tight junctions and reduced IL-8 levels [159]. Additionally, the expression of MHC class I and II molecules on DCs can be inhibited by resveratrol, thus attenuating the differentiation and maturation of DCs and subsequent failure to activate naive T cells [160]. Resveratrol also regulates adaptive immunity and aligns with the remission of gut inflammation. Studies have shown that the administration of resveratrol converts the ratio of Th1 and Th17 cells and increases the proportion of Treg cells in mouse models of IBD [107,161].

### 5.4. Other Agents

The potential modulatory properties of EGCG on the gut microbiota have been illustrated previously. EGCG can still affect oxidative stress and the immune response in IBD treatment. Notably, supplementation with EGCG can thicken the intestinal mucus and reduce intestinal permeability by enhancing tight junctions. It enhances the thickness of the mucus of colon tissue and reduces intestinal permeability in experimental colitis [115,162]. In addition, EGCG can intensify the total antioxidant capacity by activating SOD and glutathione peroxidase and attenuating the levels of MDA and NO in colon tissue [115]. In the immune response, EGCG supplementation can influence multiple immune cells, including neutrophils, macrophages, dendritic cells, and T cells. In summary, EGCG can restrict neutrophil infiltration, promote M2 macrophage polarization, weaken dendritic cell differentiation, and increase the Treg/Th17 ratio [162,163,164,165,166]. (Table 2).

## 6. Emerging Strategies Promote the Application of Polyphenols in IBD Therapy

Due to their versatility, polyphenols are increasingly used in bioimaging, therapeutic delivery, and other biomedical applications [167,168]. This is because polyphenols possess a number of desirable characteristics, including inherent biocompatibility, bioadhesion, antioxidant activity, and antibacterial activity [167]. Although numerous polyphenols have been explored in preclinical or clinical trials, their practical application in IBD treatment remains challenging due to photothermal instability, poor solubility, nonspecific selectivity, and variable oral bioavailability [169,170]. For example, polyphenols spontaneously oxidize based on their phenolic hydroxyl groups and the catechol ring [171], which leads to the loss of stability and an increasing probability of degradation [172]. Moreover, the single use of polyphenols, compared with combination with other drugs, exhibits limited therapeutic effects in cancer treatment [173]. To surmount these limitations of polyphenols, multiple strategies have been explored and developed to achieve the efficient implementation of polyphenols in IBD treatment.

### 6.1. Chemical Modification

**Figure 5:**
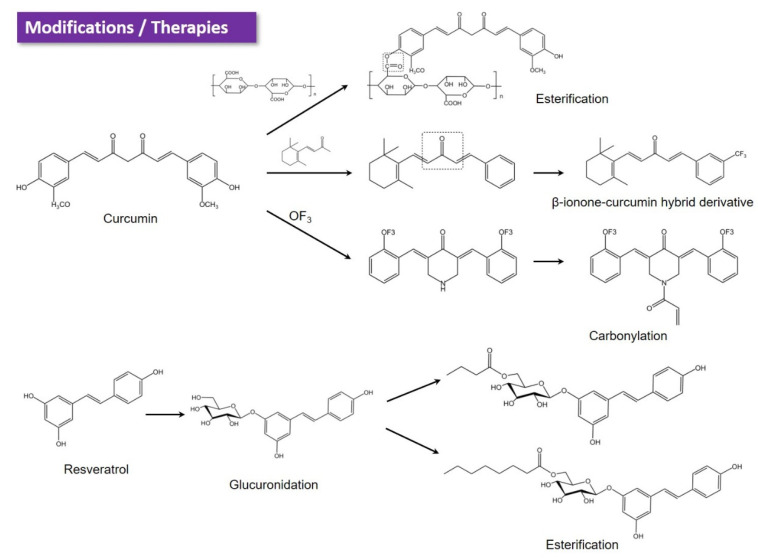
The application of the modification of polyphenols in IBD therapy. Many chemical modifications, including esterification, glycosylation, and other chemical synthetic modification, can enhance metabolic stability, water solubility, and bioavailability by changing the structure of polyphenols, thus promoting anti-inflammation and antioxidation.

Recently, structural modifications, including esterification, methylation, glycosylation, and other chemical modifications, have been developed which exhibit superior properties to circumvent the natural weakness of polyphenols and thus expand their application in antioxidant and anti-inflammatory therapy. For instance, compared to the parent curcumin, the hydrophilicity and bioavailability of the curcumin–alginate esterification products are markedly increased. The ester bond between curcumin and alginate is dissolved with the help of esterases secreted by the commensal anaerobes of the colon, releasing the antioxidant curcumin to treat UC [174]. Similarly, hybrid derivatives of β-ionone and curcumin can enhance anti-inflammatory activity by inhibiting the production of NO and ROS, thus alleviating colon length shortening and protecting against colon injury [175]. In addition, some mono-carbonyl curcumin derivatives were designed to improve curcumin’s biological activities, namely, metabolic stability, high lipid solubility, and bioavailability. For instance, ortho-substituted mono-carbonyl curcumin derivatives (2-methoxy- and 2-trifluoromethoxy- derivatives and 2-methoxy- and 2-trifluoromethoxy-substituted derivatives) demonstrated the ability to inhibit the production of RNS and ROS against oxidative and nitrosative stress in a mouse colitis model [176,177,178]. Furthermore, the benzene ring in these derivatives can be substituted by other rings, namely, the pyridine ring, furan ring, naphthalene ring, and lactam ring, and has been used to prevent and treat various diseases [179,180]. Interestingly, when compared with crude resveratrol, both resveratrol-3-O-(6′-O-butanoyl)-β-d-glucopyranoside and resveratrol-3-O-(6′-O-octanoyl)-β-d-glucopyranoside revealed better clinical efficacy in IBD treatment by slowing the metabolism of resveratrol, restoring the imbalance of the intestinal mucosal barrier, and preventing diarrhea [181]. Moreover, some glucosyl, glucosylacyl, and glucuronide derivatives of resveratrol also play a significant role in the adhesion of some intestinal bacteria by inhibiting IL-8 secretion, which can potentially be employed in the prevention of IBD [182]. Overall, the modification of polyphenols could circumvent rapid degradation and enhance their accessibility, thus providing potential strategies to facilitate the application of polyphenols in IBD therapy (Figure 5).

### 6.2. Nano-Strategies

**Figure 6:**
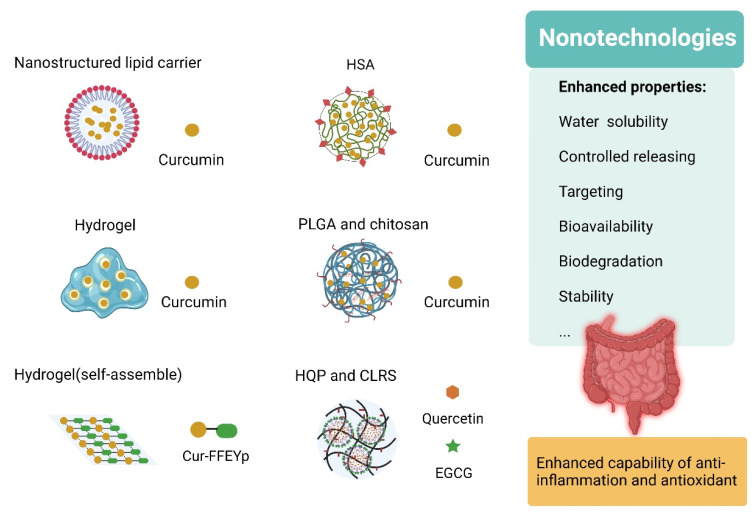
Nano-strategies in IBD treatment. Multiple nano-carriers, including nanostructured lipid carriers, degradable hydrogels, and nonimmunogenic protein-based nanocarriers, have been widely utilized to precisely deliver polyphenols. Strategies for the codelivery of polyphenols with other drugs have also been exploited to amplify the application of polyphenols in IBD treatment.

In addition to chemical modification, lipid nanocarriers and liposomes, biodegradable polymeric nanoparticles, nanoemulsions, micelles, and protein-based nanocarriers have been employed to form polyphenol-containing nanoparticles to overcome the shortcomings of polyphenols [183]. Due to their biocompatibility, biodegradability, and immunomodulatory capabilities, nanostructured lipid carriers were utilized to deliver curcumin in the treatment of IBD, displaying improved efficacy over direct administration of curcumin [184]. Similarly, another polyphenol, oleuropein, was encapsulated into nanostructured lipid carriers to make nanoparticles with improved anti-inflammatory and ROS-scavenging activity [185]. Coincidentally, in terms of the unique physical properties of hydrogels, various hydrogels have also been developed to deliver polyphenols with the aim of controllable and continuous release of packaged drugs. A pH-sensitive hydrogel has been formed from composite hyaluronic acid/gelatin containing carboxymethyl chitosan microspheres, which showed potential for the effective delivery of curcumin in the treatment of colitis [186]. In another study, instead of directly loading curcumin, Cur-FFEYp, the Cur precursor Cur–Phe–Phe–Glu–Tyr (H_2_PO_3_)–OH, was capable of local self-assembly into hydrogels via dephosphorylation and then disassembly for release by esterase in inflamed regions. This novel strategy can markedly enhance the anti-inflammatory effect of curcumin and was found to alleviate two types of IBD [187]. Human serum albumin (HSA), a U.S. FDA-approved nanomaterial, possesses various advantages, including good biocompatibility, biodegradation, water solubility, low cytotoxicity, and non-immunogenicity. Tannic acid-coated HSA was used to encapsulate curcumin via the crosslinking action of genipin, leading to increased efficiency of curcumin oral administration in treating UC [188]. In addition, codelivery of polyphenol nanoparticles and other drugs have been developed to improve IBD therapy efficiency. For instance, curcumin and siCD86 were simultaneously encapsulated by PLGA and chitosan to achieve combinational therapeutic effects for UC [189]. Interestingly, in a recent study, two different polyphenols, quercetin and epigallo-catechin 3-gallate (EGCG) were both encapsulated by hydrolytic quinoa protein (HQP) and cationic lotus root starch (CLRS) in a layer-by-layer assembly method. Quercetin was entrapped within the hydrophobic core of HQP micelles, and then EGCG bound to the hydrophilic surface, while CLRS was used to cover this carrier (Quercetin-HQP-EGCG) as a favorable coating layer, enhancing the stability and bioavailability of the resulting nanoparticle [190]. Taken together, nanostrategies can effectively address the limitations of polyphenols, improving the viability of their application in the clinical treatment of IBD (Figure 6). 

### 6.3. Combination with Other Agents

**Figure 7:**
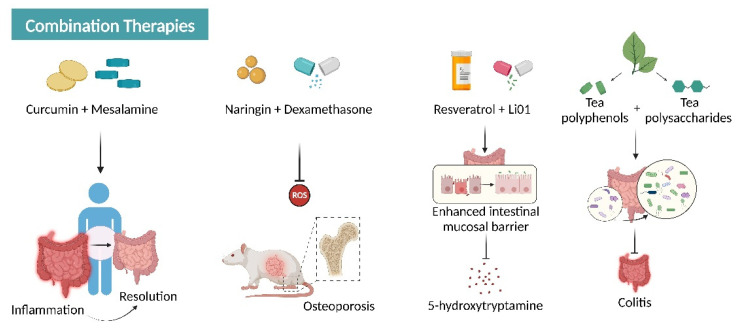
Drug combinations enhance the therapeutic effect of polyphenols in IBD. Combination strategies can exert synergistic effects and reduce side effects to facilitate the application of polyphenols in IBD.

Recent evidence has highlighted that single drug administration sometimes cannot achieve successful therapeutic goals in IBD treatment [191]. Due to the complex and diverse clinical features of IBD patients, individualized treatment based on drug combinations is gradually being applied in clinical practice. To address drug resistance and side effects in IBD therapy, an increasing number of studies have been dedicated to combining polyphenols with conventional medications. For example, based on its ability to ameliorate murine experimental colitis, curcumin combined with mesalamine, a front line treatment for mild to moderate UC, effectively maintained remission in UC patients while causing no obvious adverse effects, and outperformed the combination of placebo and mesalamine [192]. Osteoporosis, or bone loss, is a common extraintestinal side effect when treating IBD with steroids, especially glucocorticoids. A recent study found that naringin, a natural polymethoxylated flavonoid, can reverse the downregulated expression of bone formation-related genes in dexamethasone-treated IBD rats by impeding oxidative stress [193]. Interestingly, in another study, a novel strategy of combining resveratrol and *Ligilactobacillus salivarius* Li01 (Li01), a probiotic strain capable of promoting the recovery of the gut microbiota and gut barrier, showed favorable synergetic effects in treating colitis mice. Resveratrol promotes the growth of Li01 in vivo, which in turn affects the turnover of resveratrol into more bioactive metabolites, such as dihydroresveratrol, which can facilitate the suppression of inflammation by activating the environmental sensor mammalian aryl hydrocarbon receptor (AHR) and affecting the serotonin pathway [194]. In another approach, the combination of polysaccharides (TPS) with tea polyphenols (TPP) decreased the relative abundance of intestinal probiotics, including *Lactobacillaceae* and *Lactobacillus*, and thus improved the intestinal barrier function, thereby alleviating colitis [195]. In addition, other studies have focused on combining different polyphenols or combining polyphenols with other drugs in IBD therapy, such as the combination of resveratrol with melatonin and the combined application of baicalin and emodin [196,197]. Collectively, these novel strategies have the potential to eliminate the drawbacks of polyphenols per se and thus accelerate the development of polyphenol-based agents with increased anti-inflammatory ability and decreased side effects to overcome IBD (Figure 7).

## 7. Conclusions and Perspective

The pathogenesis of IBD is extremely complex and is related to the genes, environment, immune system, oxidative stress, gut microbiota, and other psychological factors. Common symptoms (e.g., abdominal pain, diarrhea, blood in the stool, malabsorption of nutrients, fatigue, and anemia) aggravate the risk of depression and anxiety [198]. In addition, persistent oxidative stress, dysbiosis, inflammation, infection, and malnutrition increase the risk of developing colorectal cancer [199]. In the clinic, the major goal of IBD treatment is to prolong the remission period and prevent a recurrence. Conventional therapeutics such as medicines (e.g., 5-aminosalicylic acid, infliximab) and steroid hormones are effective for IBD treatment. Nevertheless, these therapeutic regimens have side effects and limitations that reduce the quality of life of IBD patients. Importantly, natural polyphenols can effectively overcome these side effects and limitations in treating IBD. In this review, from the perspective of oxidative stress, inflammation, and the gut microbiota, we have summarized the corresponding potential mechanisms of pathogenesis of IBD. In addition, we have detailed several polyphenols that can function in IBD by mitigating oxidative stress, restoring gut microbial diversity, and assuaging inappropriate immune responses. We have also discussed novel strategies to drive polyphenols as ideal agents for IBD treatment, including chemical modification, nanotechnology, and combination treatment. As numerous preclinical and clinical trials have been implemented in recent years [NCT00718094, NCT03408847], the administration of polyphenols will become a novel therapeutic regimen against IBD.

## Figures and Tables

**Table 1:** Effects of polyphenols on oxidative stress and inflammation by modulation of gut microbiota in IBD.

Polyphenolic Compound	Model	Dose	Duration	Effects	References
Curcumin	type 2 diabetic mice with colitis	100 mg/kg/day	21 days	Restoring the homeostasis of Th17/Treg and improving the composition of the intestinal microbiota	[85]
	Azoxymethane-induced Il10^−/−^ mice model	8–162 mg/kg/day	15 weeks	Increasing bacterial richness, preventing age-related decrease in alpha diversity, increasing the relative abundance of *Lactobacillales*, and decreasing Coriobacterales order	[86]
	Dextran sodium sulphate (DSS)-induced colitis mice model	-	11 days	Increasing the abundance of butyrate-producing bacteria and fecal butyrate level; inhibiting the expression of inflammatory mediators; suppressing the activation of NF-κB	[87]
	DSS-induced colitis mice model	50 mg/mL or 150 mg/mL	7 days	Mitigating intestinal inflammation via inhibiting the MAPK/NFκB/STAT3 pathway; enhancing intestinal barrier and modulating abundance of some bacteria (*Akkermansia*, *Coprococcus*, etc.)	[88]
Quercetin and its metabolites	DSS-induced colitis mice model	500–1500 ppm	6 days	Upregulating transcription of GCLC to eliminate excessive ROS; inhibiting AQP3 and upregulating NOX1/2	[93]
	DSS-induced colitis mice model	10 mg/kg body weight	7 weeks	Enhancing the anti-inflammatory and bactericidal effects of macrophages via the Nrf2/HO-1 pathway; rebalancing the function of enteric macrophages	[94]
	DSS-induced colitis mice model	0.21% quercetin preparation comprising 0.15% polyphenols	8 days	Increasing the concentration of MPO, GSH, MDA, NO; revising the decrease in Chao1 and ACE	[95]
Quercetin and its metabolites	Citrobacter rodentium-induced colitis mouse model	-	2 weeks	Enhancing the populations of *Bacteroides*, *Bifidobacterium*, *Lactobacillus*, and Clostridia and reducing those of *Fusobacterium* and *Enterococcus*; suppressing the production of pro-inflammatory cytokines	[96]
	DSS-induced ulcerative colitis rat model	10 mg/kg	5 days	Inhibiting COX-2 and iNOS protein expression; increasing levels of pro-inflammatory cytokines in the plasma	[99]
Resveratrol	DSS-induced ulcerative colitis rat model	100 mg/kg/day	10 days	Reducing paracellular permeability and the secretion of proinflammatory cytokines and upregulating tight junction proteins via AMPK-mediated activation of CDX2 and the regulation of the SIRT1/NF-κB pathway	[103]
	DSS-induced colitis in pseudo-germ-free mice	100 mg/kg	19 days	Attenuating the inflammatory response by regulating MAPK and NF-κB pathways	[106]
	2,4,6-trinitrobenzenesulfonic acid (TNBS)-induced colitis mice model	100 mg/kg	5 days	Increasing the production of i-butyric acid; ameliorating imbalance of gut microbiota induced by TNBS; inducing Tregs while suppressing inflammatory Th1/Th17 cells	[107]
	AOM/DSS-induced CRC mice model	100 mg/kg	10 weeks	Inhibiting histone deacetylases (HDACs); inducing Tregs while suppressing inflammatory Th1/Th17 cells	[108]
	DSS-induced ulcerative colitis rat model	1 mg/kg/day	25 days	Increasing lactobacilli and bifidobacteria and diminishing the increase in enterobacteria	[109]
protocatechuic acid	TNBS-induced colitis in mice	30 mg/kg and 60 mg/kg	5 days	Decreasing oxidized/reduced glutathione ratio; increasing the expression of Nrf2 and inhibiting the expression of proinflammatory cytokines	[100]
EC, EGC, ECG, EGCG, etc.	TNBS-induced colitis rat model	_	14 days	Downregulating the expression of TNF-α, NF-κB, IL-1β, and IL-6; resisting oxidative stress via both non-canonical and canonical NF-kB pathway	[114]
EGCG	DSS-induced ulcerative colitis mice model	50 mg/kg	3 days	Increasing SCFAs-producing bacteria, such as *Akkermansia*	[115]
Ellagic acid and pomegranate extract (PE)	TNBS-induced colitis rat model	10 mg/kg/day	2 weeks	Reducing MPO activity and the TNF-α levels; alleviating COX-2 and iNOS overexpression, reducing MAPKs phosporylation and preventing the nuclear NF-κB translocation	[116]
Pomegranate peel extract	Citrobacter rodentium-induced colitis mice model	0.2 mL twice a day	2 weeks	Decreasing the Firmicutes/Bacteroidetes ratio, increasing the abundance of *Proteobacteria* and *Verrucomicrobiae*	[117]

**Table 2:** Polyphenols regulate the immune response and oxidative stress in IBD.

Polyphenolic Compound	Target	Effects	References
Curcumin	APC	Impairing ROS (H2O2)-induced oxidative damage by stimulating the heme oxygenase-1 (HO-1) signaling pathway	[147]
	naive T cells, T_CM_, T_EM_	Downregulating the levels of proinflammatory cytokines such as IL-7, IL-15, and IL-21 by inhibiting the JAK1/STAT5 signaling pathway	[148]
	Tfh, Tfr	Correcting the imbalance in Tfh and Tfr through the inhibition of IL-21	[149]
	Macrophage	Changing macrophage polarization from M1 to M2 and decrease the expression of PRRs such as TLR2, TLR4, and NF-κB	[150]
Quercetin	Treg cell, Macrophage	Reducing significantly gut inflammation, increase Treg cells and reduce Th17 cells	[152]
	Intestinal goblet cell	Regulating the secretory function of intestinal goblet cells and mucin levels via acting PKCα/ERK1-2 signal pathway	[153]
		Promoting the synthesis of GSH and the expression of Nrf2 to alleviate oxidative stress	[95]
	Macrophage	Promoting M2 macrophage polarization to decrease the secretion of proinflammatory cytokines such as TNF-α, IL-23, and IL-12 in colonic tissue	[94]
	Dendritic cells	Affecting C/EBP-β to inhibit the production of downstream cytokines, including TNF-α and IL-6 in dendritic cells, thereby attenuating colitis	[155]
Resveratrol	Intestinal epithelial cell	Decreasing the production of the inflammatory cytokine tumor necrosis factor-α, interleukin-6, and interleukin-1β; increasing tight junction proteins occludin and ZO-1	[157]
	Gut epithelial cell	Reversing the inflammatory effects of TNF-α by reducing IL-1β and increasing IL-11 production	[158]
	Neutrophil	Attenuating the recruitment and infiltration of neutrophils into colon tissue, which may be attributed to enhanced tight junctions and reduced IL-8 levels	[159]
	DCs	Inhibiting the expression of MHC class I and II molecules on DCs, thus attenuating the differentiation and maturation of DCs and subsequent failure to activate naive T cells	[160]
	Treg cell, Th17 cell	Converting the ratio of Th1 and Th17 cells and increasing the proportion of Treg cells in mouse models of IBD	[161]
EGCG	Mucus epithelial cell	Enhancing the thickness of the mucus epithelial cells of colon tissue and reduce intestinal permeability in experimental colitis	[162]
	Neutrophils, macrophages, dendritic cells, and T cells	Restricting neutrophil infiltration, promoting M2 macrophage polarization, weakening dendritic cell differentiation, and increasing the Treg/Th17 ratio	[163,164,165]

## Data Availability

Not applicable.

## Abbreviations

AHR: Aryl hydrocarbon receptor; AOM: Azoxymethane; APCs: Antigen-presenting cells; CD: Crohn’s disease; CHM: Chinese herbal medicine; CLRS: Cationic lotus root starch; COX-2: cyclooxygenase-2; C/EBP-β: CCAAT/enhancer binding protein β; DAMPs: Damage-associated molecular patterns; DCs: Dendritic cells; DSS: Dextran sulfate sodium; EA: Ellagic acid; EGCG: Epigallocatechin-3-gallate; FXR: Farnesoid X receptor; GPCRs: G protein-coupled receptors; GSH: Glutathione; HIF-1α: Hypoxia-inducible factor-1α; HO-1: Heme oxygenase-1; HOCl: Hypochlorous acid; HSA: Human serum albumin; HQP: Hydrolytic quinoa protein; HSPs: Heat shock proteins; IBD: Inflammatory bowel disease; IECs: Intestinal epithelial cells; iNOS: Inducible nitric oxide synthase; Li01: *Ligilactobacillus salivarius Li01*; LPS: Lipopolysaccharide; MDA: Malondialdehyde; MPO; Myeloperoxidase; NETs: Neutrophil traps; NODs: Nucleotide oligomerization domains; NOX: NADPH Oxidases; PAMPs: Pathogen-associated molecular patterns; PRRs: Pattern recognition receptors; ROS: Reactive oxygen species; SCFAs: short-chain fatty acids; SOD: Superoxide dismutase; TCM: traditional Chinese medicine; T_CM_: Central memory T cells; T_EM_: Effector memory T cells; Tfh: Follicular helper T cells; Tfr: Follicular regulatory T cells; TGR5: Takeda G protein-coupled receptor 5; TLR: Toll-like receptors; TNBS: 2,4,6-trinitrobenzenesulfonic acid; TPP: Tea polyphenols; TPS: Tea polysaccharides; Tregs: T regulatory cells; UC: Ulcerative colitis.
